# Improved Leg Tracking Considering Gait Phase and Spline-Based Interpolation during Turning Motion in Walk Tests

**DOI:** 10.3390/s150922451

**Published:** 2015-09-04

**Authors:** Ayanori Yorozu, Toshiki Moriguchi, Masaki Takahashi

**Affiliations:** 1 School of Science for Open and Environmental Systems, Graduate School of Science and Technology, Keio University, 3-14-1 Hiyoshi, Kohoku-ku, Yokohama 223-8522, Japan; 2 Research & Development Division, Murata Machinery, Ltd., 136 Takeda-Mukaishiro-cho, Fushimi-ku, Kyoto 612-8686, Japan; E-Mail: toshiki.moriguchi@drw.muratec.co.jp; 3 Department of System Design Engineering, Keio University, 3-14-1 Hiyoshi, Kohoku-ku, Yokohama 223-8522, Japan; E-Mail: takahashi@sd.keio.ac.jp

**Keywords:** gait measurement, timed up and go, laser range sensor, Kalman filter, data association, spline-based interpolation

## Abstract

Falling is a common problem in the growing elderly population, and fall-risk assessment systems are needed for community-based fall prevention programs. In particular, the timed up and go test (TUG) is the clinical test most often used to evaluate elderly individual ambulatory ability in many clinical institutions or local communities. This study presents an improved leg tracking method using a laser range sensor (LRS) for a gait measurement system to evaluate the motor function in walk tests, such as the TUG. The system tracks both legs and measures the trajectory of both legs. However, both legs might be close to each other, and one leg might be hidden from the sensor. This is especially the case during the turning motion in the TUG, where the time that a leg is hidden from the LRS is longer than that during straight walking and the moving direction rapidly changes. These situations are likely to lead to false tracking and deteriorate the measurement accuracy of the leg positions. To solve these problems, a novel data association considering gait phase and a Catmull–Rom spline-based interpolation during the occlusion are proposed. From the experimental results with young people, we confirm that the proposed methods can reduce the chances of false tracking. In addition, we verify the measurement accuracy of the leg trajectory compared to a three-dimensional motion analysis system (VICON).

## 1. Introduction

**Figure 1:**
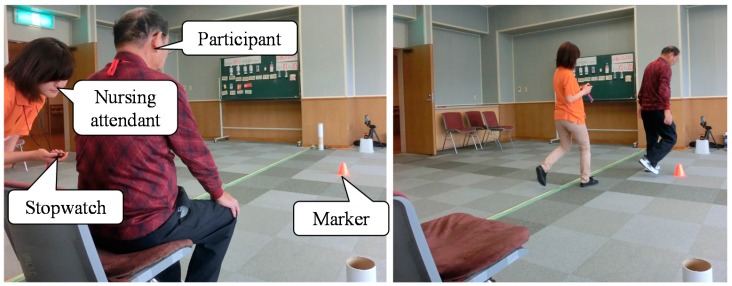
Appearance of the timed up and go test (TUG) measurement in actual community health centers.

Falling is a leading cause of unintentional injury and death in the elderly [1,2] and can also result in impaired mobility, disability, fear of falling and reduced quality of life [3,4,5]. Unsurprisingly, the prevention of falls in the elderly is a public health priority in many countries across the world [6,7,8]. Falling is a common problem in the growing elderly population, and there is a need for effective and convenient fall-risk assessment tools that can be used in community-based fall prevention programs. There are many tests that assess the motor function of the elderly. In particular, the timed up and go test (TUG) is the clinical test most often applied to evaluate elderly individual ambulatory ability in many clinical institutions and local communities [9]. It is also listed as one of the main measurement tests in a guide to physical fitness for the elderly [10]. In the TUG, as shown in Figure 1, a participant rises from a chair, walks three meters, turns around a marker, walks back to the chair and sits down. The participant is instructed to perform the TUG at maximum walking speed. To evaluate the fall risk of the participant, walking parameters, such as walking speed (m/s), cadence (step/s), stride length (m), step length (m) and step width (m), are used. To measure these walking parameters for the evaluation of the risk of falling from the TUG, a measurement system that can measure the trajectory of both legs across several meters is required.

In many cases, force plates [11,12] or three-dimensional motion measuring devices, such as the VICON system [13,14], have been used to measure the walking parameters with high reliability. However, because of their cost, scale and lack of convenience, it is difficult to install these devices in community health centers. Therefore, as shown in Figure 1, since the measurement of the effects of this training is carried out by observation using a stopwatch in actual community health centers, it is difficult to measure the walking parameters for fall-risk assessment of the participant.

Much research has used wearable IMUs for assessing clinical tasks [15,16,17,18]. As a non-contact measurement system, an ultrasonic sensor, a laser range sensor (LRS) [19] or an RGB-depth sensor, such as the Microsoft Kinect [20], can be used. These devices are comparatively small and inexpensive. Several methods of tracking people’s center of gravity using these devices have been proposed [21,22,23,24,25,26]. In [21], a sonar-sensor-based walking human detection method has been proposed. In [22], a pedestrian tracking system using multiple mobile robots equipped with an LRS at the trunk height has been proposed. In [23,24], people tracking with the sample-based joint probabilistic data association filter for mobile robot navigation has been proposed. In [25], a people detection and tracking method using an LRS and a camera has been proposed. To detect humans, the LRS was installed at shin height, and the leg detection method based on three observed leg patterns from LRS data has been proposed. In [26], a robust multi-person tracking based on the histograms of oriented gradients, like classification using the RGB-depth data, has been proposed. To measure the walking parameters, the system has to track both legs and obtain their positions. Several methods to obtain the posture and the lower limbs of a pedestrian based on the RGB-depth data have also been proposed [27,28,29,30]. To measure walking parameters in several meter walk tests, such as the TUG, the sensor must be able to obtain high accuracy distance data over a wide range. In this study, we develop a gait measurement system using an LRS, one of the non-contact measurement systems, because it is necessary to assess many participants in a short time in actual community health centers. The LRS is a comparatively small and inexpensive device and can obtain highly accurate two-dimensional distance data over a wide range. To measure the walking parameters, the LRS is installed at shin height, and the system detects and tracks both legs of the participant. A method used to track both legs and to measure walking parameters based on the LRS data has been proposed and verified in straight walk tests [31,32]. Previously, we proposed a leg detection method with five observed leg patterns and global nearest neighbor (GNN)-based [33] data association considering the state of each leg [34] to reduce losing track of the leg and the false tracking for several meter walk tests, such as the multi-target stepping task. In [34], since the sampling time is sufficiently shorter than the gait cycle time, a constant velocity motion model is given. In [35], a biped walking model with a walking frequency has been proposed. In the biped walking model, a constant velocity motion model is given for both legs’ tracking. In [36], four gait phases assuming the acceleration and deceleration on the swing leg were defined, and a simplified acceleration motion model based on the gait phases has been proposed. Since participants attempt to perform the TUG at maximum walking speed, the change of the leg speed in the TUG is generally larger than in normal walking. Therefore, in this study, an acceleration model taking gait phase into account [36] is used.

**Figure 2:**
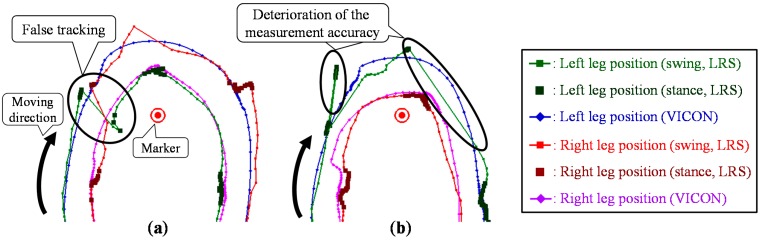
Top view of the leg trajectories and the problems to be solved for the TUG measurement. (**a**) False tracking. (**b**) Deterioration of the measurement accuracy during the occlusion.

Particularly during the turning motion in the TUG, both legs might be close to each other, and one leg might be hidden from the sensor. In addition, the moving direction of the leg changes rapidly because of the turning motion. Furthermore, LRS distance data are likely to be disturbed during the turning motion, and the observed leg positions might not be correctly calculated using the leg patterns. Figure 2 shows the leg trajectories measured by the LRS system and the VICON system. As shown in Figure 2a, these situations are likely to lead to false tracking via switching of the left and right legs. Moreover, if the leg is unobservable from the LRS, the leg position is obtained as the position predicted based on the state equation of the Kalman filter. In the turning motion, the time that a leg is hidden from the LRS is longer than that in straight walking, and the moving direction of the leg rapidly changes. As shown in Figure 2b, the situation is likely to lead to deterioration in the measurement accuracy of the leg positions. To improve leg tracking in these situations, a novel data association considering gait phase and a Catmull-Rom spline-based [37] interpolation during the occlusion are proposed.

To verify the effectiveness of the proposed method, experiments with seven young people were carried out. The trajectory of both legs at the LRS height acquired by the proposed system were compared to the result measured using a three-dimensional motion analysis system (VICON).

## 2. System Overview

### 2.1. Configuration

**Figure 3:**
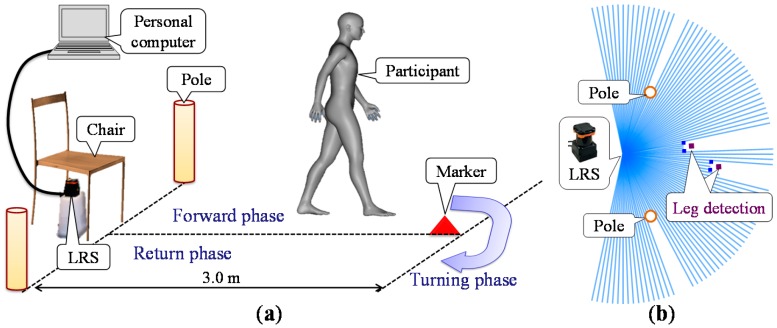
Gait measurement system using an LRS for the TUG. (**a**) System configuration. (**b**) Image of the LRS scan data.

Figure 3a shows a proposed gait measurement system for the TUG. The system consists of an LRS, a personal computer, a marker and two calibration poles. As shown in Figure 3a, three TUG phases (forward, turning and return phases) are defined to verify the measurement accuracy in each TUG phase. 

**Figure 4:**
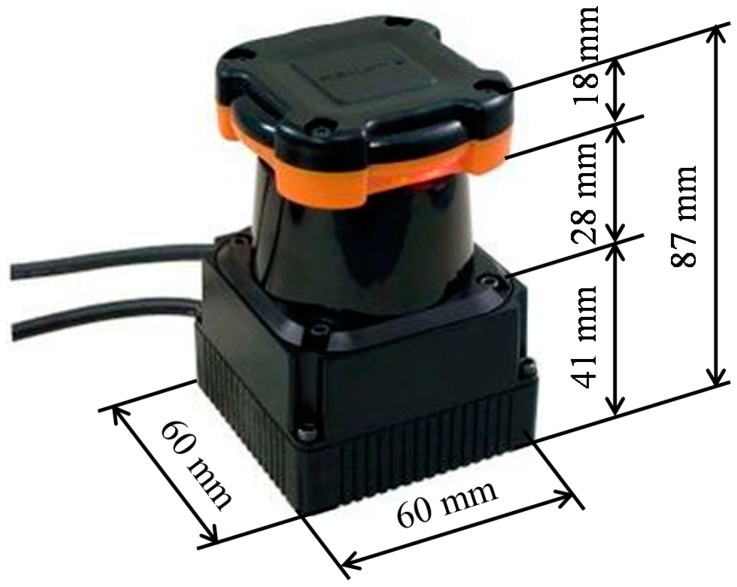
Appearance of the UTM-30LX.

In the system, the LRS is installed at shin height (0.27 m in our experimental system) and captures distance data by scanning a single laser beam in a horizontal plane, as shown in Figure 3b. In this study, as shown in Figure 4, the UTM-30LX (Hokuyo Automatic Co, Ltd., Osaka, Japan [19]) was used. Table 1 shows the specification of the UTM-30LX. The UTM-30LX has a scanning range from −135°–135° in steps of 0.25° (0° is in the front of the device), and one scan is completed in 0.025 s (40 Hz). The UTM-30LX was attached to the personal computer using a USB 2.0 port. In this study, the personal computer was a Panasonic Let’s note LX3 with a 2.1-GHz Intel Core i7-4600U processer. The raw scan data provided by the device contain 1081 distance data expressed in millimeters and represented using 18 bits with a total of 3243 bytes per scan.

**Table 1:** Specifications of the UTM-30LX.

Parameters	Values
Laser Wavelength	905 nm, Class 1
Power Source	12 V ± 10%
Current Consumption	0.7 A, max 1.0 A
Detection Range	0.1–30 m, max 60 m
Measurement Accuracy	0.1–10 m: ±0.03 m 10–30 m: ±0.05 m
Scan Angle	270°
Angular Resolution	0.25° (360°/1440)
Scan Time	25 ms (40 Hz)
Interface	USB 2.0
Weight	0.233 kg

### 2.2. Algorithm

**Figure 5:**
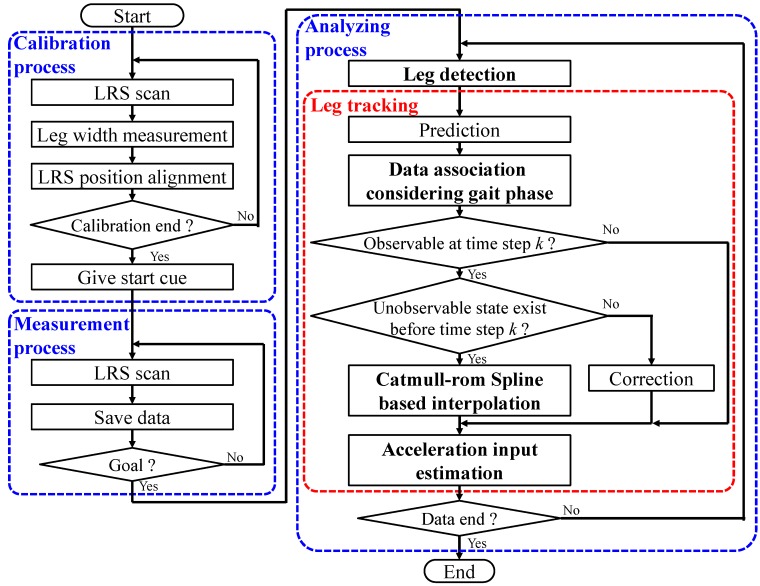
Algorithm of the gait measurement system using a laser range sensor (LRS) for the TUG.

As shown in Figure 5, the system consists of three main processes. In the calibration process before TUG measurement, the system measures the leg width $w_{l}$ of the participant at shin height and aligns the field and the LRS using two poles as described in [38]. In the TUG measurement process, the system scans and saves LRS distance data. In the gait analysis process, after the participant finishes the TUG, the system detects the legs using the saved LRS scan data and tracks both legs using the Kalman filter, taking into account the gait phase and interpolation based on the Catmull–Rom spline during the occlusion. Section 3 presents the detail of the human walking model for the Kalman filter. Section 4 presents the leg detection. Section 5 presents the detail of the leg tracking taking into account the gait phase and interpolation based on the Catmull–Rom spline during the occlusion.

## 3. Human Walking Model

**Figure 6:**
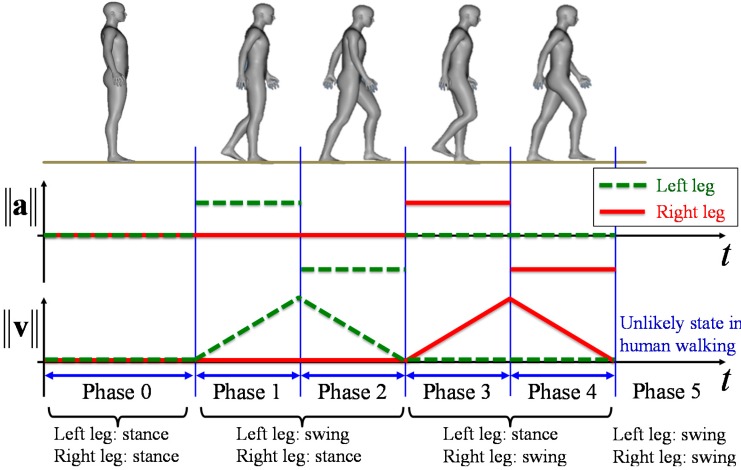
A simplified human walking model.

During normal human walking, one leg swings by pivoting on the other one. The role of each leg alternates by landing and moving in shifts in a rhythmic pattern. Zhao and Shibasaki proposed a simplified acceleration walking model for steady walking [36]. At the start and end of the TUG, the participant is stationary. To take into account the stationary state, as shown in Figure 6, an extended walking model, including the state where both legs are in the stance phase and swing phase, is proposed. Six gait phases are defined as follows. Phase 0 is the state where both legs are in the stance phase. Phase 1 is the state where the left leg is accelerating in the swing phase and the right leg is in the stance phase. Phase 2 is the state where the left leg is decelerating in the swing phase and the right leg is in the stance phase. Phase 3 is the state where the left leg is in the stance phase and the right leg is accelerating in the swing phase. Phase 4 is the state where the left leg is in the stance phase and the right leg is decelerating in the swing phase. Finally, Phase 5 is the unlikely state in human walking where both legs are in the swing phase. The gait phase can be identified based on the positional relationship between the legs and velocity.

### 3.1. State Equation of the Kalman Filter

Leg position and velocity is estimated using the Kalman filter with an acceleration motion model considering the gait phase. The discrete time model of leg motion is given as follows:

(1) $$x_{k}^{f}=Ax_{k-1}^{f}+B_{u}u_{k-1}^{f}+B\text{Δ}x_{k-1}^{f}\;\left(f=L,\;R\right)$$

where $\text{A}=\left[\begin{array}{cccc} 1 & 0 & \text{Δ}t & 0\\ 0 & 1 & 0 & \text{Δ}t\\ 0 & 0 & 1 & 0\\ 0 & 0 & 0 & 1 \end{array}\right],\;B_{u}\text{=B}=\left[\begin{array}{cc} \text{Δ}t^{2}/2 & 0\\ 0 & \text{Δ}t^{2}/2\\ \text{Δ}t & 0\\ 0 & \text{Δ}t \end{array}\right]$ and $x_{k}^{f}={\left[\begin{array}{cccc} x_{k}^{f} & y_{k}^{f} & {\dot{x}}_{k}^{f} & {\dot{y}}_{k}^{f} \end{array}\right]}^{T}$. $\left(x_{k}^{f},\;y_{k}^{f}\right):={\text{p}}_{k}^{f}$ is the estimated position and $\left({\dot{x}}_{k}^{f},\;{\dot{y}}_{k}^{f}\right):={\text{v}}_{k}^{f}$ is the estimated velocity of the leg (f=L, R, where *L* and *R* indicate the left and right legs, respectively). $u_{k}^{f}={\left[\begin{array}{cc} {\ddot{x}}_{k}^{f} & {\ddot{y}}_{k}^{f} \end{array}\right]}^{T}$ is the acceleration input vector corresponding to the gait phase. $\text{Δ}{\text{x}}_{k}^{f}={\left[\begin{array}{cc} n^{{\dot{x}}_{k}} & n^{{\dot{y}}_{k}} \end{array}\right]}^{T}$ is the acceleration disturbance vector, which is assumed to be zero mean and has a white noise sequence with covariance Q. Δt is a sampling time. Then, the measurement model is as follows:

(2) $$y_{k}^{f}=\text{C}{\text{x}}_{k}^{f}+{\text{w}}_{k}$$

where $\text{C}=\left[\begin{array}{cccc} 1 & 0 & 0 & 0\\ 0 & 1 & 0 & 0 \end{array}\right]$. ${\text{w}}_{k}={\left[\begin{array}{cc} n^{x_{k}} & n^{y_{k}} \end{array}\right]}^{T}$ is the measurement noise, which is assumed to be zero mean and has a white noise sequence with covariance R.

### 3.2. Gait Phase Identification

From validation compared with a force plate [38], it is possible to identify the gait phase (in the stance phase or swing phase) considering the speed of both legs in human walking. The condition where the right leg is in the stance phase is:

(3) $$\left\|{\text{v}}_{k}^{R}\right\|<\left\|{\text{v}}_{k}^{L}\right\|\;\vee \;\left\|{\text{v}}_{k}^{R}\right\|<v_{st\_th}$$

The condition where the right leg is in the swing phase is:

(4) $$\left\|{\text{v}}_{k}^{R}\right\|>\left\|{\text{v}}_{k}^{L}\right\|\;\vee \;\left\|{\text{v}}_{k}^{R}\right\|>v_{sw\_th}$$

where $v_{st\_th}$ and $v_{sw\_th}$ are the thresholds of the maximum speed in the stance phase and the minimum speed in the swing phase, respectively. The gait phase of the left leg is identified in the same way. Then, the gait phase is identified considering the relative positional relationship of both legs and velocity. First, if both legs are in the stance phase, the gait phase is identified as Phase 0. Second, if the left leg is in the swing phase and the right leg is in the stance phase, the gait phase is identified based on the inner product of the velocity vector of the left leg and the relative position vector of the right leg from the left leg:

(5) $$\left(p_{k}^{R}-p_{k}^{L}\right)\cdot v_{k}^{L}$$

If Equation (5) has a positive value, the gait phase is identified as Phase 1. Otherwise, the gait phase is identified as Phase 2. If the left leg is in the stance phase and the right leg is in the swing phase, the gait phase is identified in the same way. Finally, if both legs are in the swing phase, the gait phase is defined as Phase 5.

## 4. Leg Detection

The observed leg positions ${\text{y}}_{k}^{j}\left(j=1,\cdot \cdot \cdot ,J\right)$ are calculated based on the leg width $w_{l}$ and five observed leg patterns [34]. 

First, to calculate the leg positions, the system searches for edges $e_{m}^{h}\left(m=1,\cdot \cdot \cdot ,M_{k}\right)$ from the LRS scan data using the following equation:

(6) $$\left|l_{i}-l_{i+1}\right|>w_{l}/2$$

where $l_{i}$ is the *i*-th laser-scanned distance data from the right of an LRS. Moreover, the detected edges are identified by $e_{m}^{B}=i\text{​},\;e_{m+1}^{F}=i+1$ when $l_{i}>l_{i+1}$ and $e_{m}^{F}=i,\;e_{m+1}^{B}=i+1$ when $l_{i}<l_{i+1}$ (h=F, B, where *F* and *B* indicate the forward and backward edges, respectively). $M_{k}$ is the total number of detected edges at time step k.

**Figure 7:**
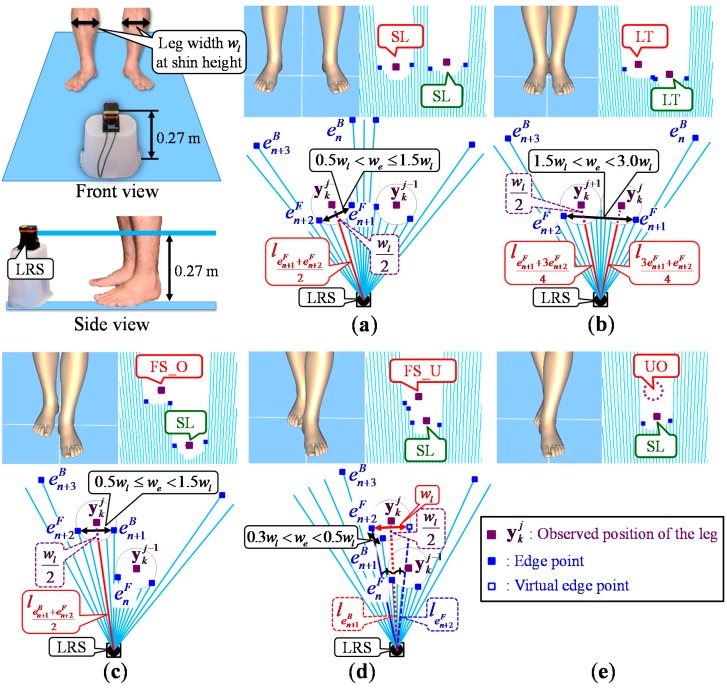
Leg detection using five observed leg patterns [34]. (**a**) single leg (SL) pattern; (**b**) legs together (LT) pattern; (**c**) forward straddle observable (FS_O) pattern; (**d**) forward straddle unobservable (FS_U) pattern; (**e**) unobservable (UO) pattern.

Then, the system calculates the observed leg positions ${\text{y}}_{k}^{j}\left(j=1,\cdot \cdot \cdot ,J\right)$ considering five observed leg patterns based on their spatial relationship and the width $w_{e}$ between the edges. As shown in Figure 7, the five observed leg patterns are SL (single leg), LT (legs together), FS_O (forward straddle observable), FS_U (forward straddle unobservable) and UO (unobservable).

## 5. Leg Tracking

This study presents an improved leg-tracking method using a novel data association taking into account periodic gait phase changes and the Catmull–Rom spline-based [37] interpolation during the occlusion.

### 5.1. Prediction

**Figure 8:**
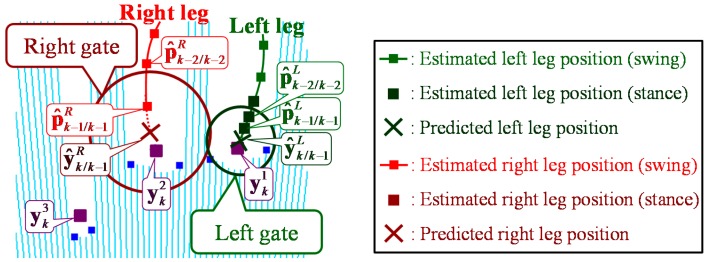
Data association with gates.

As shown in Figure 8, based on the model of leg motion, the system predicts the position of the tracked leg by:

(7) $${\hat{y}}_{k/k-1}^{f}=\text{C}{\hat{\text{x}}}_{k/k-1}^{f}=\text{C}\left(\text{A}{\hat{\text{x}}}_{k-1/k-1}^{f}+B_{u}u_{k-1}^{f}\right)$$

where ${\hat{\text{x}}}_{k/k-1}^{f}$ and ${\hat{\text{x}}}_{k-1/k-1}^{f}$ are the *a priori* state estimate at time step k and the *a posteriori* state estimate at the time step k−1. In addition, *a priori* covariance matrix $P_{k/k-1}^{f}$ is calculated by:

(8) $$P_{k/k-1}^{f}=\text{A}P_{k-1/k-1}^{f}{\text{A}}^{T}+QBQ^{T}$$

where $P_{k-1/k-1}^{f}$ is the *a posteriori* covariance matrix at the time step k−1.

### 5.2. Data Association Considering Gait Phase

As shown in Figure 8, to eliminate unlikely observation-to-track associations, a validation region (gate) is constructed around the predicted position [39]. The simplest and most widely-spread approach for data association is GNN [33]. In the GNN data association, if observations are included in the gate, the observation-to-track association is always executed. However, during the turning motion in the TUG, both legs might be close to each other, and one leg might be hidden from the sensor. Furthermore, LRS distance data are likely to be disturbed during the turning motion, so the observed leg positions might not be correctly calculated using the leg patterns. In these situations, the gate is likely to include other leg’s observation or misdetections. These situations are likely to lead to false tracking using the GNN data association. To deal with these situations, we propose a novel data association taking into account periodic gait phase changes. 

The following cost function is defined for all observation-to-track associations:

(9) $$c_{a,b}=d_{L,a}+d_{R,b}\;(0\leq a\leq J,0\leq \;b\leq J,\;a\neq b)$$

The element $d_{f,j}$ of the cost function is the matching cost between the predicted position ${\hat{y}}_{k/k-1}^{f}$ of the tracked leg and the *j*-th observed position ${\text{y}}_{k}^{j}$ and has the following values:

(10) $$d_{f,j}=\{\begin{array}{c} \lambda_{f,j}\\ d_{max} \end{array}\;\begin{array}{c} if\;y_{k}^{j}\;\mathrm{is}\;\mathrm{in}\;\mathrm{the}\;\mathrm{gate}\;\mathrm{of}\;{\hat{y}}_{k/k-1}^{f}\\ if\;y_{k}^{j}\text{ is not in the gate of }{\hat{y}}_{k/k-1}^{f}\text{ or }j=0 \end{array}$$

where j=0 indicates a false alarm. $\lambda_{f,j}$ is the Mahalanobis distance and is calculated as follows:

(11) $$\lambda_{f,j}=\sqrt{{\left(y_{k}^{j}-{\hat{y}}_{k/k-1}^{f}\right)}^{T}{\left(S_{k}^{f}\right)}^{-1}\left(y_{k}^{j}-{\hat{y}}_{k/k-1}^{f}\right)}$$

where $S_{k}^{f}$ is the covariance of the innovation $\left(y_{k}^{j}-{\hat{y}}_{k/k-1}^{f}\right)$:

(12) $$S_{k}^{f}=CP_{k/k-1}^{f}C^{T}+R$$

Then, $y_{k}^{j}$ is in the gate of ${\hat{y}}_{k/k-1}^{f}$ according to:

(13) $${\lambda_{f,j}}^{2}<G$$

where G is a gate and can be determined from the chi-square $\left(\chi^{2}\right)$ distribution with two degrees of freedom in the system.

In all observation-to-track associations, the filtering process of the Kalman filter is performed and the gait phase is identified. The association whose cost function is minimized is selected from among the associations whose gait phase is unlikely to change from time step k−1. The unlikely changes of gait phase in human walking are as follows:

∙ Phase 0 to Phase 5,
∙ Phase 1 to Phases 0, 3, 4 and 5,
∙ Phase 2 to Phases 1, 4 and 5,
∙ Phase 3 to Phases 0, 1, 2 and 5,
∙ Phase 4 to Phases 2, 3 and 5.

### 5.3. Correction

If the corresponding observation $y_{k}^{f}$ exists, a filtering (correction) process of the Kalman filter is performed. *A posteriori* state estimate ${\hat{\text{x}}}_{k/k}^{f}$ is calculated by:

(14) $${\hat{\text{x}}}_{k/k}^{f}={\hat{\text{x}}}_{k/k-1}^{f}+K_{k}^{f}\left(y_{k}^{f}-C{\hat{\text{x}}}_{k/k-1}^{f}\right)$$

where $K_{k}^{f}$ is a Kalman gain:

(15) $$K_{k}^{f}=P_{k/k-1}^{f}C^{T}{\left(CP_{k/k-1}^{f}C^{T}+R\right)}^{-1}$$

Then, *a posteriori* covariance matrix $P_{k/k}^{f}$ is calculated by:

(16) $$P_{k/k}^{f}=\left(I-K_{k}^{f}C\right)P_{k/k-1}^{f}$$

If no corresponding observation exists (UO leg pattern), the correction is not performed. The *a posteriori* state estimate and covariance matrix are set to the *a priori* state estimate and covariance matrix, and the system continues the tracking.

### 5.4. Catmull–Rom Spline-Based Interpolation during the Occlusion

If the leg is unobservable from the LRS, the leg position is obtained as the position predicted based on the state equation of the Kalman filter. In the TUG turning phase, the time that a leg is hidden from the LRS is longer than that in the TUG straight walking (forward and return) phase, and the moving direction of the leg is changed. As shown in Figure 2b, the situation is likely to lead to deterioration in the measurement accuracy of the leg positions. To deal with the situation, a spline-based interpolation during the occlusion is proposed.

**Figure 9:**
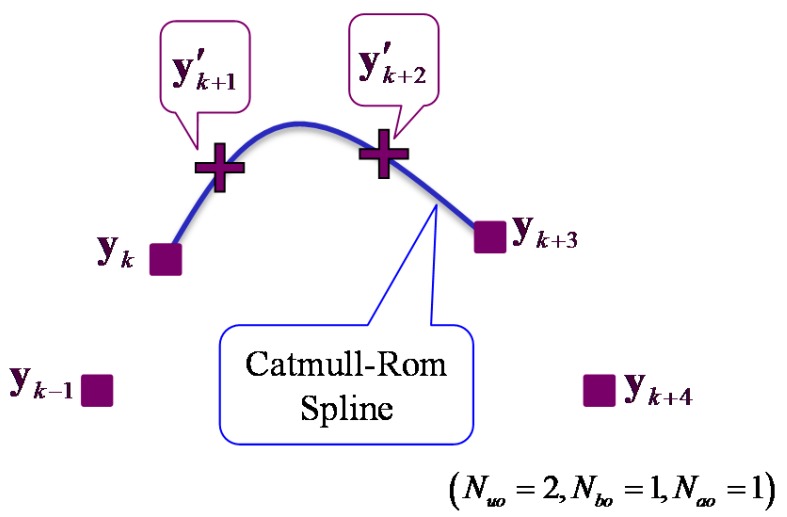
Vertical observations during the occlusion are calculated by the Catmull–Rom spline.

First, in the proposed interpolation, the observations during the occlusion are calculated virtually based on the Catmull–Rom spline to obtain a smooth trajectory from the four passing points [37]. As shown in Figure 9, if the $N_{uo}$-steps leg positions from time step to $k+N_{uo}+1$ are unobserved, vertical observations $y{'}_{k+1}$ to $y{'}_{k+N_{uo}}$ are calculated using four observations $y_{k-N_{bo}}$, $y_{k}$ before the occlusion and $y_{k+N_{uo}+1}$, $y_{k+N_{uo}+N_{ao}+1}$ after the occlusion:

(17) $$\begin{array}{l} {y^{\prime }}_{k+i}=\frac{1}{2}\left[1\;\tau \;\tau^{2}\;\tau^{3}\right]\left[\begin{array}{cccc} 0 & 2 & 0 & 0\\ -1 & 0 & 1 & 0\\ 2 & -5 & 4 & -1\\ -1 & 3 & -3 & 1 \end{array}\right]\left[\begin{array}{c} y_{k-N_{bo}}\\ y_{k}\\ y_{k+N_{uo}+1}\\ y_{k+N_{uo}+N_{ao}+1} \end{array}\right]\\ \tau =\frac{i}{N_{uo}+1}\;\left(i=1,\cdots ,N_{uo}\right) \end{array}$$

Then, to consider the human walking model, the prediction and filtering (correction) process of the Kalman filter are performed again using the vertical observations from time step k, and all state parameters are updated. Improvements of not only the measurement accuracy during the occlusion, but also the leg tracking performance can be expected by the proposed interpolation.

### 5.5. Acceleration Input Estimation

In Phase 0, where both legs are in the stance phase, the acceleration input vector $u_{k}^{f}$ is:

(18) $$u_{k}^{L}=0,\;u_{k}^{R}=0$$

In Phase 1, where the left leg is accelerating in the swing phase and the right leg is in the stance phase, $u_{k}^{f}$ is:

(19) $$u_{k}^{L}=g_{k}^{L}v_{k}^{L}/\left\|v_{k}^{L}\right\|,\;u_{k}^{R}=0$$

Then, in Phase 2, where the left leg is decelerating in the swing phase and the right leg is in the stance phase, $u_{k}^{f}$ is:

(20) $$u_{k}^{L}=-g_{k}^{L}v_{k}^{L}/\left\|v_{k}^{L}\right\|,\;u_{k}^{R}=0$$

$g_{k}^{f}$ is the acceleration function that is calculated as the average of the norm of the acceleration vector $\left({\dot{x}}_{k}^{f}/\text{Δ}t,\;{\dot{y}}_{k}^{f}/\text{Δ}t\right):=a_{k}^{f}$ in the swing phase of the previous $N_{ac}$ steps. In Phases 3 and 4, $u_{k}^{f}$ is calculated in the same way.

## 6. Experiments

### 6.1. Experimental Conditions

To verify the effectiveness of the proposed method, seven young volunteers (six men, one woman, mean age 23.0 ± 1.9 years) were recruited as participants for this study. Each participant performed the TUG four times (a total of 28 trials). The leg trajectory measured using the proposed system was compared to those measured using a three-dimensional motion analysis system (VICON) with seven cameras. 

**Figure 10:**
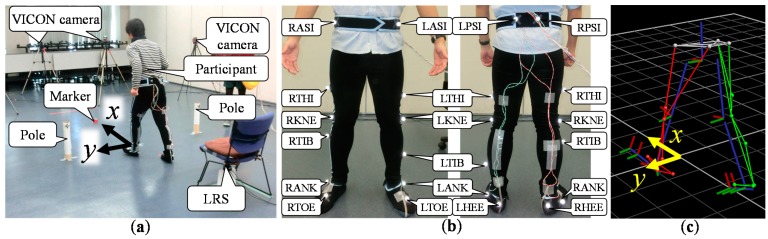
(**a**) Environmental field. (**b**) VICON markers were attached to the participant.(**c**) VICON analysis with the plug-in-gait model.

Figure 10a shows the field of the TUG. The field coordinate of the proposed system was fixed to that of VICON by using two poles. As shown in Figure 10b,c, VICON markers were attached to 16 places on the lower limbs of the participant, and the plug-in-gait model [14] was used for motion analysis. In VICON analysis, the leg trajectory was calculated as the trajectory at the LRS height. The sampling time of the VICON system was 5.0 ms (200 Hz). To verify the measurement accuracy of the proposed system, the leg trajectory measured by the proposed system was compared to those measured using the VICON system. 

**Table 2:** Proposed system parameters.

Parameters	Values
Sampling Time Δ*t*	25 ms (40 Hz)
Motion Covariance **Q**	diag(15.0^2^, 15.0^2^)
Observation Covariance **R**	diag(0.04^2^, 0.04^2^)
Threshold of the Speed in the Stance Phase *v_st_th_*	0.47
Threshold of the Speed in the Swing Phase *v_sw_th_*	0.93
Gate *G* (Probability *P_G_* = 0.999)	13.82
Number of Steps for the Acceleration Function *N_ac_*	40

Table 2 shows the values of the proposed system parameters. To verify the effectiveness of the proposed leg tracking method with the data association considering gait phase and the Catmull–Rom spline-based interpolation during the occlusion, three methods, labelled 1–3 (see Table 3 for definitions), were used. In Method 1, GNN data association [33] was used. In Methods 2 and 3, the proposed data association considering the gait phase was used. In Method 3, the proposed interpolation during the leg occlusion was used. 

### 6.2. Experimental Results

**Figure 11:**
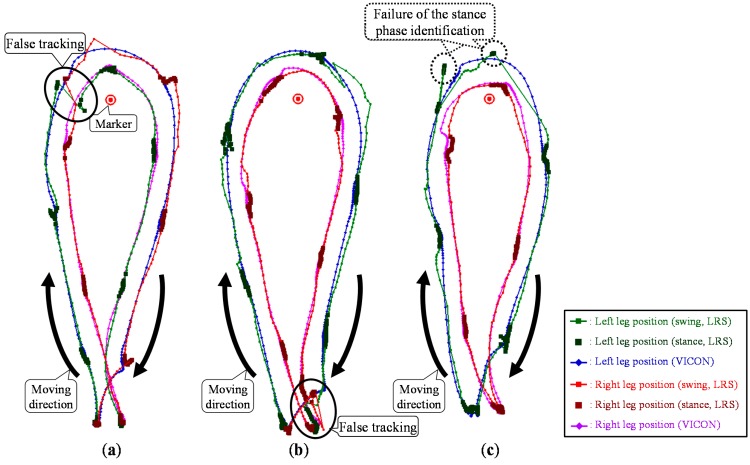
Leg tracking results of false tracking and the failure of the stance phase identification. (**a**) False tracking via switching of the left and right legs during turning motion with Method 1. (**b**) False tracking during walking back to the chair with Method 2 (**c**) Failure of the stance phase identification during the turning motion with Method 2.

To measure the walking parameters, the system should track both legs without false tracking via switching of the left and right legs and identify the stance phase to calculate foot contact positions. In this study, successful leg tracking was defined as the system tracking both legs without false tracking and identifying the stance phase correctly. Figure 11 shows the leg tracking results of false tracking and the failure of the stance phase identification. 

Table 3 shows the leg tracking success rate (tracking success number/total 28 trials) and the number of false tracking and failures of the stance phase identification of each method. As shown in Table 3, the tracking success rate of the comparison method with the GNN data association (Method 1) was 50.0% (14/28). In Method 1, the false tracking was likely to occur during the turning motion, as shown in Figure 11a. In addition, the failure of the stance phase identification occurred during the turning motion, as shown in Figure 11c. Secondly, the tracking success rate of the proposed data association considering the gait phase (Method 2) was 89.3% (25/28). Method 2 reduced the amount of false tracking during the turning motion. As shown in Figure 11b, the occlusion and changing moving direction also occurred at the end of TUG (during walking back to chair) and led to false tracking. The failure of the stance phase identification also occurred during the turning motion. Finally, the tracking success rate of the proposed method with data association considering gait phase and interpolation (Method 3) was 96.4% (27/28). From the experimental results, it was confirmed that the proposed leg tracking method with data association considering gait phase and the Catmull–Rom spline-based interpolation could reduce the amount of false tracking. Then, we verify the effectiveness of the proposed data association and interpolation in Section 6.3 and Section 6.4. 

**Table 3:** Definitions and leg tracking results of each method of a total of 28 trials.

		Method 1	Method 2	Method 3 (Proposal)
Data Association Considering Gait Phase		No	Yes	Yes
Catmull–Rom Spline-based Interpolation		No	No	Yes
Number of False Tracking	During Turning Motion	12	0	0
	During Walking Back to Chair	0	2	1
Number of Failure of the Stance Phase Identification During Turning Motion		2	1	0
Tracking Success Rate		50.0% (14/28)	89.3% (25/28)	96.4% (27/28)

### 6.3. Effectiveness of the Data Association Considering the Gait Phase

**Figure 12:**
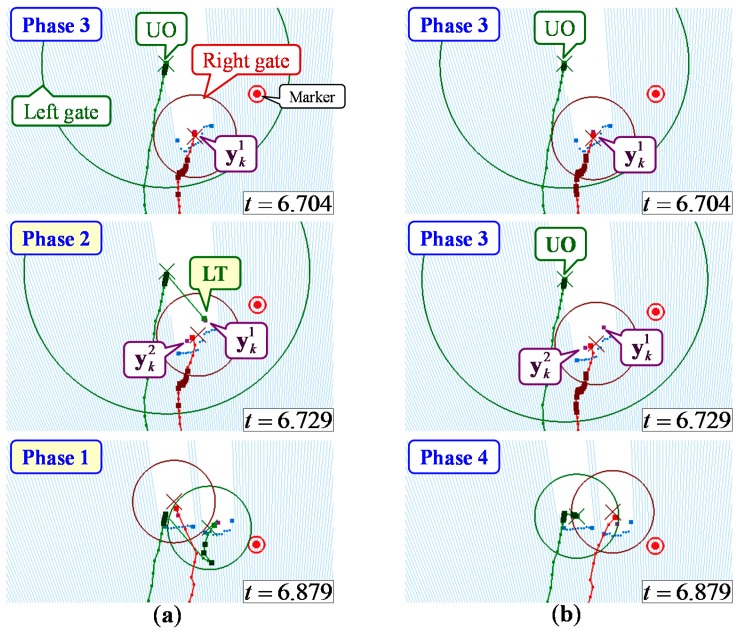
Leg tracking result in a situation where the left leg was temporarily hidden and LRS distance data were disturbed. (**a**) Method 1: global nearest neighbor (GNN) data association. (**b**) Method 2: data association considering gait phase.

As shown in Table 3, false tracking was likely to occur during the turning motion in the TUG. Figure 12 shows the example of the leg tracking results of Methods 1 and 2 in the situation where the left leg was temporarily hidden from the LRS and LRS distance data were disturbed during the turning motion. As shown in Figure 12, the left leg was hidden by the right leg at time *t* = 6.704 and the left gate expanded. Then, two observed leg positions were detected at time *t* = 6.729. However, the observed position $y_{k}^{1}$ was a misdetection because of the disturbed distance data. In the GNN data association (Method 1), if the observed position was included in the gate, the observation-to-track association was always executed. Therefore, in this situation, false tracking via switching of the left and right legs occurred. However, in the proposed data association (Method 2), taking into account periodic gait phase changes, a $y_{k}^{1}$-to-left leg and $y_{k}^{2}$-to-right leg association was identified as a false alarm because a gait phase change from three to two was unlikely in human walking. Then, the system successfully tracked both legs without false tracking at time *t* = 6.879. As shown in Table 3, the tracking success rate of the proposed data association (Method 2) was improved compared to that of Method 1. It was confirmed that the proposed data association could reduce the amount of false tracking during the turning motion in the TUG.

### 6.4. Effectiveness of the Catmull–Rom Spline-Based Interpolation

**Figure 13:**
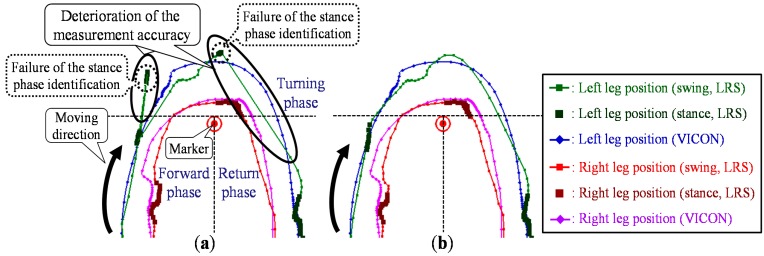
Leg tracking result in a situation where the left leg was hidden. (**a**) Method 2: without interpolation. (**b**) Method 3: with interpolation.

Figure 13 shows the example of the leg tracking results of Methods 2 and 3 in the situation where the left leg was hidden from the LRS. Table 4 shows the occlusion rate (unobservable time steps/total time steps) and RMSE (root mean squared error) of the leg trajectory at the LRS height compared to VICON analysis in each TUG phase of the tracking success data of Methods 2 and 3. If the leg was unobservable from the LRS, the leg position was obtained as the position predicted based on the state equation of the Kalman filter. As shown in Table 4, particularly in the TUG turning phase, the time that a leg was hidden from the LRS was longer than that in the TUG straight walking (forward and return) phase, and the moving direction of the leg was rapidly changed. Therefore, as shown in Table 4 and Figure 13a, the measurement accuracy of the left leg was deteriorated in the TUG turning phase. In addition, as shown in Figure 13a, the deterioration of the measurement accuracy during the occlusion leads to the failure of the stance phase identification.

**Table 4:** Occlusion rate and RMSE of the leg trajectory in each TUG phase of the tracking success data of Methods 2 and 3.

TUG Phase	Method 2 (without Interpolation) 25 Tracking Success Data			Method 3 (Proposal: with Interpolation) 27 Tracking Success Data		
	Occlusion Rate	RMSE (m)		Occlusion Rate	RMSE (m)	
		*x*-direction	*y*-direction		*x*-direction	*y*-direction
Forward	3.5% (225/6364)	0.042	0.018	3.8% (264/6912)	0.042	0.019
Turning	11.3% (255/2264)	0.069	0.062	12.3% (304/2462)	0.055	0.049
Return	5.1% (344/6686)	0.053	0.028	5.5% (402/7294)	0.047	0.025
Total	5.4% (824/15314)	0.051	0.032	5.8% (970/16668)	0.047	0.028

Table 5 shows the RMSE of the leg trajectory at the LRS height in each leg observation state compared to VICON analysis of the success tracking data of Methods 2 and 3. As shown in Table 5 and Figure 13b, the proposed interpolation improved the measurement accuracy during the occlusion. Therefore, as shown in Table 4, the measurement accuracy in the TUG turning phase was improved by the proposed interpolation. In addition, as shown in Table 3, the tracking success rate of the proposed method with interpolation (Method 3) was improved compared to that of Method 2. It was confirmed that the proposed interpolation could also reduce the amount of false tracking. From the experimental results, it was confirmed that the proposed interpolation improved leg tracking performance and the measurement accuracy of the leg during the turning motion. 

**Table 5:** RMSE of the leg trajectory in each leg observation state of the tracking success data of Methods 2 and 3.

Leg Observation State	Method 2 (without Interpolation) 25 Tracking Success Data			Method 3 (Proposal: with Interpolation) 27 Tracking Success Data		
	Time Steps	RMSE (m)		Time Steps	RMSE (m)	
		*x*-direction	*y*-direction		*x*-direction	*y*-direction
Observable	14490	0.047	0.027	15698	0.045	0.025
Unobservable	824	0.102	0.081	970	0.066	0.052

**Figure 14:**
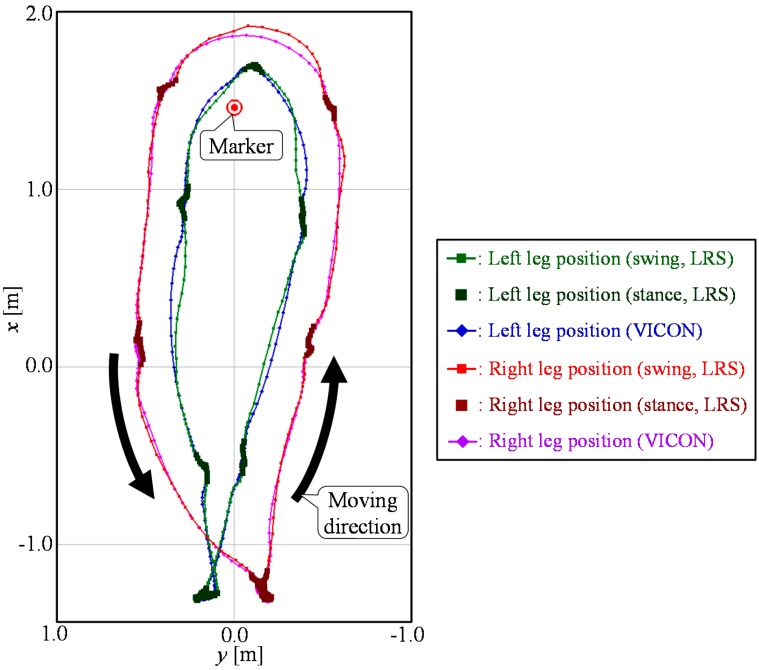
Example of the gait measurement results of the proposed method (Method 3).

Figure 14 shows an example of the gait measurement results using the proposed method (Method 3). As shown in Table 4, it was confirmed that the proposed method could measure the leg trajectory with high accuracy compared to the measurement accuracy of the LRS.

The gait measurement accuracy and range of the proposed system depend on the measurement accuracy and the angular resolution of the LRS. According to the specification shown in Table 1, the accuracy expected in a single distance measured with the UTM-30LX is ±0.03 m within a range up to 10 m and ±0.05 m in the range from 10–30 m. However, the number of measurement points available in one leg is relative to the distance from the LRS. As shown in Figure 7, at least three measurement points are required for the leg detection. To detect the leg, the relation of three parameters, the leg width $w_{l}$ of the participant, the angular resolution φ of the LRS and maximum gait measurement range $R_{max}$, of the proposed system is satisfied as follows:

(21) $$3R_{max}\frac{\pi }{180}\varphi <w_{l}$$

In addition, the accuracy of the observed leg position tends to be deteriorated, so that there are few measurement points. If $w_{l}$ is assumed to be 0.10 m, the effective gait measurement range $R_{max}$ of UTM-30LX is limited to 8.0 m [31]. In this study, we applied the proposed system to the TUG, in which a participant walks three meters from the LRS. Moreover, the proposed system can apply to other several meter walk tests considering maximum gait measurement range. Therefore, the proposed system may be helpful for community-based fall prevention programs.

## 7. Conclusions

This study presents an improved leg tracking method using a laser range sensor (LRS) for a gait measurement system to evaluate the motor function in walk tests, such as the timed up and go test (TUG). Particularly, during the turning motion in the TUG, the time that a leg is hidden from the LRS is longer than that in straight walking, and the moving direction rapidly changes. These situations are likely to lead to false tracking and deteriorate the measurement accuracy of the leg positions. To solve these problems, a novel data association taking periodic gait phase changes into account and a Catmull–Rom spline-based interpolation during the occlusion were proposed.

From the experimental results with several young people, it was confirmed that the proposed leg tracking method considering gait phase reduced the chance of false tracking. It was also confirmed that the proposed interpolation during the occlusion improved leg tracking performance and the measurement accuracy of the leg. In addition, we verified the measurement accuracy of the leg trajectory compared to a three-dimensional motion analysis system (VICON).
